# Ethyl *ent*-15α-[(2-nitro­benz­yloxy)meth­yl]-16-oxobeyeran-20-oate

**DOI:** 10.1107/S1600536812029418

**Published:** 2012-07-04

**Authors:** Ya Wu, Xia Wang, Jian-hong Gong, Jing-chao Tao

**Affiliations:** a Pharmacy College, Henan University of Traditional Chinese Medicine, Zhengzhou 450008, People’s Republic of China; b Department of Chemistry, New Drug Research & Development Center, Zhengzhou University, Zhengzhou 450052, People’s Republic of China

## Abstract

In the title compound, C_30_H_41_NO_6_, the three six-membered rings adopt chair conformations and the stereochemistry of the *A*/*B* and *B*/*C* ring junctions are *trans*. The five-membered ring *D* adopts an envelope conformation, with the methyl­ene C atom as the flap. The title compound was synthesized *via* esterification, Tollens reaction, 1,5-hydride shift from the natural tetracyclic diterpenoid isosteviol

## Related literature

For related structures, see: Wu *et al.* (2009 ▶, 2012 ▶). For the biological activity of the tetra­cyclic diterpenoid isosteviol (*ent*-16-ketobeyeran-19-oic acid) and its derivatives, see: Chang *et al.* (2008 ▶); Li *et al.* (2011 ▶); Liu *et al.* (2001 ▶); Roy *et al.* (2007 ▶); Wong *et al.* (2006 ▶); Yasukawa *et al.* (2002 ▶).
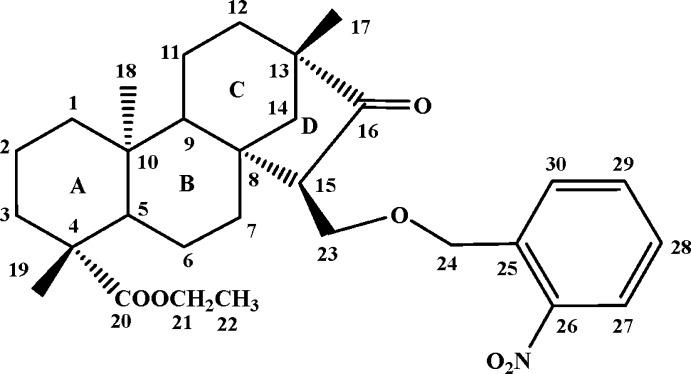


## Experimental

### 

#### Crystal data

C_30_H_41_NO_6_
*M* *_r_* = 511.64
Orthorhombic, 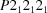

*a* = 8.3711 (17) Å
*b* = 11.419 (2) Å
*c* = 28.685 (6) Å
*V* = 2742.0 (10) Å^3^
*Z* = 4
Mo *K*α radiation
μ = 0.09 mm^−1^
*T* = 291 K
0.20 × 0.18 × 0.17 mm

#### Data collection

Oxford Diffraction Xcalibur Eos Gemini diffractometer
Absorption correction: multi-scan (*CrysAlis PRO*; Oxford Diffraction, 2010 ▶) *T*_min_ = 0.983, *T*_max_ = 0.986
7952 measured reflections
2688 independent reflections
2350 reflections with *I* > 2σ(*I*)
*R*_int_ = 0.056

#### Refinement

*R*[*F* ^2^ > 2σ(*F* ^2^)] = 0.070
*wR*(*F* ^2^) = 0.186
*S* = 1.10
2688 reflections
335 parameters
H-atom parameters constrained
Δρ_max_ = 0.18 e Å^−3^
Δρ_min_ = −0.17 e Å^−3^

### 

Data collection: *CrysAlis PRO* (Oxford Diffraction, 2010 ▶); cell refinement: *CrysAlis PRO*; data reduction: *CrysAlis PRO*; program(s) used to solve structure: *SHELXS97* (Sheldrick, 2008 ▶); program(s) used to refine structure: *SHELXL97* (Sheldrick, 2008 ▶); molecular graphics: *SHELXTL* (Sheldrick, 2008 ▶); software used to prepare material for publication: *SHELXTL*.

## Supplementary Material

Crystal structure: contains datablock(s) global, I. DOI: 10.1107/S1600536812029418/wn2481sup1.cif

Structure factors: contains datablock(s) I. DOI: 10.1107/S1600536812029418/wn2481Isup2.hkl

Additional supplementary materials:  crystallographic information; 3D view; checkCIF report

## Figures and Tables

**Fig. 1.**
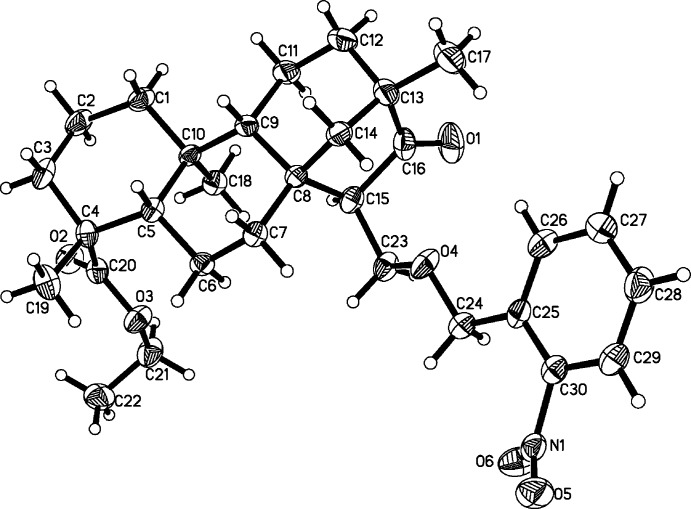
View of the title compound, with displacement ellipsoids drawn at the 30% probability level. Hydrogen atoms are shown as spheres of arbitrary radius.

**Fig. 2.**
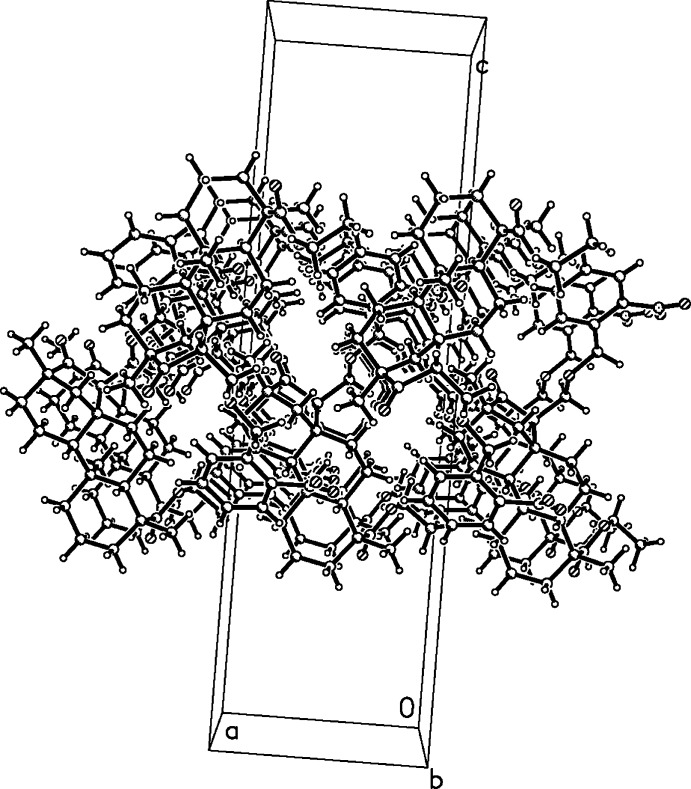
A view, along the b axis, of the crystal packing.

## supplementary crystallographic information

### Comment

The natural tetracyclic diterpenoid isosteviol (*ent*-16-ketobeyeran-19-oic acid) and its derivatives have a remarkably broad spectrum of biological activities including anti-inflammatory (Yasukawa *et al.*, 2002), glucocorticoid agonist (Chang *et al.*, 2008), antihypertension (Liu *et al.*, 2001), antitumor (Li *et al.*, 2011), antiproliferation (Wong *et al.*, 2006) and inhibition of *ent*-kaurene synthase (Roy *et al.*, 2007). The title compound was synthesized *via* esterification, Tollens reaction, 1,5-hydride shift from isosteviol in order to develop a new antitumor for therapeutic use. The crystal structures of some related compounds have already been published (Wu *et al.*, 2009, 2012).

In the title compound the three six-membered rings adopt chair conformations and the stereochemistry of the A/B and B/C ring junctions are *trans*. The five-membered ring D adopts an envelope conformation, with atom C14 displaced from the C8/C15/C16/C13 plane by 0.172 (2) Å.

### Experimental

To a stirred solution of ethyl-*ent*-15α-hydroxymethyl-16β-hydroxybeyeran -20-oate (0.378 g, 1 mmol) and ο-nitrobenzaldehyde (0.167 g, 1.1 mmol) in acetonitrile (8 mL) was added sulfuric acid (0.1 mmol). After stirring for 4 h at room temperature, the mixture was concentrated under vacuum and extracted with CHCl_3_ and H_2_O. The organic compound was washed with a saturated aqueous solution of NaCl, dried with MgSO_4_ and concentrated under vacuum. The residue was purified by column chromatography on silica (petroleum ether/ethyl acetate 6:1, *v*/*v*) to give the product C_30_H_41_NO_6_ (0.429 g, 84%). Colorless single crystals of the title compound suitable for X-ray analysis were obtained by slow evaporation of an acetone solution.

### Refinement

Anomalous dispersion was negligible and Friedel pairs were merged before refinement. H atoms were positioned geometrically and refined as riding atoms with Csp^2^—H = 0.93 Å, Cmethyl—H = 0.96 Å, Cmethylene—H = 0.97 Å, Cmethine—H = 0.98 Å; U_iso_(H) = xU_eq_(C), where x = 1.5 for methyl H and 1.2 for all other H atoms.

### Crystal data

**Table:**

C_30_H_41_NO_6_	*D*_x_ = 1.239 Mg m^−^^3^
*M**_r_* = 511.64	Mo *K*α radiation, λ = 0.71073 Å
Orthorhombic, *P*2_1_2_1_2_1_	Cell parameters from 399 reflections
*a* = 8.3711 (17) Å	θ = 2–25.0°
*b* = 11.419 (2) Å	µ = 0.09 mm^−^^1^
*c* = 28.685 (6) Å	*T* = 291 K
*V* = 2742.0 (10) Å^3^	PRISMATIC, colorless
*Z* = 4	0.20 × 0.18 × 0.17 mm
*F*(000) = 1104	

### Data collection

**Table:**

Oxford Diffraction Xcalibur Eos Gemini diffractometer	2688 independent reflections
Radiation source: fine-focus sealed tube	2350 reflections with *I* > 2σ(*I*)
Graphite monochromator	*R*_int_ = 0.056
Detector resolution: 0 pixels mm^-1^	θ_max_ = 25.0°, θ_min_ = 1.4°
Oscillation frames scans	*h* = −9→9
Absorption correction: multi-scan (*CrysAlis PRO*; Oxford Diffraction, 2010)	*k* = −13→0
*T*_min_ = 0.983, *T*_max_ = 0.986	*l* = −33→33
7952 measured reflections	

### Refinement

**Table:**

Refinement on *F*^2^	Secondary atom site location: difference Fourier map
Least-squares matrix: full	Hydrogen site location: inferred from neighbouring sites
*R*[*F*^2^ > 2σ(*F*^2^)] = 0.070	H-atom parameters constrained
*wR*(*F*^2^) = 0.186	*w* = 1/[σ^2^(*F*_o_^2^) + (0.107*P*)^2^ + 0.9805*P*] where *P* = (*F*_o_^2^ + 2*F*_c_^2^)/3
*S* = 1.10	(Δ/σ)_max_ < 0.001
2688 reflections	Δρ_max_ = 0.18 e Å^−^^3^
335 parameters	Δρ_min_ = −0.17 e Å^−^^3^
0 restraints	Extinction correction: *SHELXTL* (Sheldrick, 2008), Fc^*^=kFc[1+0.001xFc^2^λ^3^/sin(2θ)]^-1/4^
Primary atom site location: structure-invariant direct methods	Extinction coefficient: 0.006 (2)

### Special details

**Table:**

Geometry. All e.s.d.'s (except the e.s.d. in the dihedral angle between two l.s. planes) are estimated using the full covariance matrix. The cell e.s.d.'s are taken into account individually in the estimation of e.s.d.'s in distances, angles and torsion angles; correlations between e.s.d.'s in cell parameters are only used when they are defined by crystal symmetry. An approximate (isotropic) treatment of cell e.s.d.'s is used for estimating e.s.d.'s involving l.s. planes.
Refinement. Refinement of *F*^2^ against ALL reflections. The weighted *R*-factor *wR* and goodness of fit *S* are based on *F*^2^, conventional *R*-factors *R* are based on *F*, with *F* set to zero for negative *F*^2^. The threshold expression of *F*^2^ > σ(*F*^2^) is used only for calculating *R*-factors(gt) *etc*. and is not relevant to the choice of reflections for refinement. *R*-factors based on *F*^2^ are statistically about twice as large as those based on *F*, and *R*- factors based on ALL data will be even larger.

### Fractional atomic coordinates and isotropic or equivalent isotropic displacement parameters (Å^2^)

**Table:**

	*x*	*y*	*z*	*U*_iso_*/*U*_eq_	
O1	0.1611 (7)	0.3605 (5)	0.97148 (15)	0.0846 (16)	
O2	0.6339 (6)	0.2134 (3)	1.23213 (12)	0.0575 (11)	
O3	0.7511 (5)	0.2638 (3)	1.16532 (11)	0.0556 (11)	
O4	0.5036 (5)	0.4970 (3)	0.96537 (11)	0.0542 (11)	
O5	0.9724 (5)	0.6750 (4)	0.86124 (15)	0.0677 (13)	
O6	0.8962 (6)	0.4991 (4)	0.87463 (16)	0.0717 (13)	
C1	0.2466 (7)	0.4154 (5)	1.19284 (16)	0.0486 (14)	
H1A	0.2225	0.4977	1.1973	0.058*	
H1B	0.1464	0.3747	1.1878	0.058*	
C2	0.3243 (8)	0.3678 (5)	1.23739 (17)	0.0553 (16)	
H2A	0.2549	0.3828	1.2638	0.066*	
H2B	0.3382	0.2837	1.2347	0.066*	
C3	0.4854 (9)	0.4252 (5)	1.24562 (16)	0.0534 (16)	
H3A	0.5335	0.3911	1.2732	0.064*	
H3B	0.4690	0.5079	1.2517	0.064*	
C4	0.6014 (7)	0.4119 (4)	1.20465 (15)	0.0411 (12)	
C5	0.5184 (7)	0.4587 (4)	1.15953 (14)	0.0366 (11)	
H5A	0.4927	0.5403	1.1670	0.044*	
C6	0.6193 (6)	0.4677 (4)	1.11497 (15)	0.0376 (11)	
H6A	0.7240	0.4988	1.1226	0.045*	
H6B	0.6337	0.3902	1.1017	0.045*	
C7	0.5394 (6)	0.5462 (4)	1.07967 (15)	0.0368 (12)	
H7A	0.6065	0.5510	1.0522	0.044*	
H7B	0.5309	0.6244	1.0927	0.044*	
C8	0.3731 (6)	0.5052 (4)	1.06500 (15)	0.0351 (11)	
C9	0.2718 (6)	0.4759 (5)	1.10952 (15)	0.0390 (12)	
H9A	0.2510	0.5521	1.1240	0.047*	
C10	0.3516 (6)	0.4019 (4)	1.14892 (15)	0.0347 (11)	
C11	0.1045 (7)	0.4302 (6)	1.09511 (18)	0.0580 (17)	
H11A	0.1147	0.3510	1.0832	0.070*	
H11B	0.0363	0.4274	1.1224	0.070*	
C12	0.0253 (7)	0.5071 (8)	1.0579 (2)	0.074 (2)	
H12A	−0.0139	0.5782	1.0724	0.089*	
H12B	−0.0658	0.4658	1.0450	0.089*	
C13	0.1400 (7)	0.5402 (6)	1.01787 (18)	0.0572 (16)	
C14	0.2821 (7)	0.6015 (5)	1.03878 (16)	0.0439 (13)	
H14A	0.2482	0.6628	1.0600	0.053*	
H14B	0.3483	0.6358	1.0146	0.053*	
C15	0.3684 (7)	0.4026 (5)	1.02853 (16)	0.0436 (13)	
H15A	0.3588	0.3280	1.0452	0.052*	
C16	0.2121 (8)	0.4254 (6)	1.00117 (17)	0.0539 (15)	
C17	0.0516 (9)	0.6066 (9)	0.9796 (2)	0.092 (3)	
H17A	−0.0371	0.5606	0.9689	0.138*	
H17B	0.1230	0.6213	0.9541	0.138*	
H17C	0.0133	0.6797	0.9918	0.138*	
C18	0.3684 (7)	0.2712 (4)	1.13629 (18)	0.0451 (13)	
H18A	0.2648	0.2389	1.1300	0.068*	
H18B	0.4165	0.2300	1.1619	0.068*	
H18C	0.4345	0.2634	1.1091	0.068*	
C19	0.7519 (9)	0.4865 (6)	1.2151 (2)	0.0624 (17)	
H19A	0.8030	0.4577	1.2428	0.094*	
H19B	0.7212	0.5667	1.2196	0.094*	
H19C	0.8247	0.4813	1.1893	0.094*	
C20	0.6598 (7)	0.2851 (5)	1.20244 (16)	0.0433 (13)	
C21	0.8085 (9)	0.1435 (5)	1.15962 (19)	0.0641 (18)	
H21A	0.8388	0.1311	1.1274	0.077*	
H21B	0.7227	0.0895	1.1669	0.077*	
C22	0.9494 (9)	0.1178 (6)	1.1906 (2)	0.0694 (19)	
H22A	0.9824	0.0380	1.1862	0.104*	
H22B	0.9198	0.1297	1.2225	0.104*	
H22C	1.0360	0.1692	1.1827	0.104*	
C23	0.5060 (8)	0.3940 (5)	0.99388 (17)	0.0490 (14)	
H23A	0.6069	0.3885	1.0104	0.059*	
H23B	0.4939	0.3247	0.9747	0.059*	
C24	0.6239 (7)	0.4964 (5)	0.93124 (18)	0.0495 (14)	
H24A	0.6267	0.4210	0.9157	0.059*	
H24B	0.7272	0.5100	0.9456	0.059*	
C25	0.5875 (7)	0.5928 (5)	0.89623 (15)	0.0428 (13)	
C26	0.4326 (8)	0.6335 (6)	0.89139 (19)	0.0576 (16)	
H26A	0.3529	0.6038	0.9106	0.069*	
C27	0.3939 (8)	0.7177 (6)	0.8584 (2)	0.0614 (17)	
H27A	0.2893	0.7444	0.8561	0.074*	
C28	0.5086 (9)	0.7620 (6)	0.82890 (19)	0.0622 (17)	
H28A	0.4812	0.8167	0.8063	0.075*	
C29	0.6655 (8)	0.7244 (5)	0.83320 (17)	0.0496 (14)	
H29A	0.7453	0.7552	0.8143	0.060*	
C30	0.7004 (7)	0.6404 (4)	0.86617 (16)	0.0398 (12)	
N1	0.8701 (6)	0.6027 (4)	0.86772 (14)	0.0447 (11)	

### Atomic displacement parameters (Å^2^)

**Table:**

	*U*^11^	*U*^22^	*U*^33^	*U*^12^	*U*^13^	*U*^23^
O1	0.100 (4)	0.095 (4)	0.059 (2)	−0.034 (3)	−0.024 (3)	−0.007 (2)
O2	0.074 (3)	0.044 (2)	0.054 (2)	0.007 (2)	0.004 (2)	0.0114 (18)
O3	0.071 (3)	0.050 (2)	0.0460 (18)	0.022 (2)	0.0126 (19)	0.0083 (16)
O4	0.074 (3)	0.047 (2)	0.0411 (17)	0.011 (2)	0.0228 (19)	0.0073 (16)
O5	0.052 (3)	0.075 (3)	0.076 (3)	−0.024 (3)	0.006 (2)	−0.005 (2)
O6	0.062 (3)	0.063 (3)	0.090 (3)	0.011 (2)	0.020 (3)	0.003 (2)
C1	0.047 (3)	0.055 (3)	0.043 (3)	0.002 (3)	0.015 (2)	0.005 (2)
C2	0.065 (4)	0.062 (4)	0.040 (3)	0.009 (3)	0.018 (3)	0.011 (2)
C3	0.082 (4)	0.046 (3)	0.032 (2)	0.012 (3)	0.005 (3)	−0.004 (2)
C4	0.051 (3)	0.036 (3)	0.036 (2)	−0.003 (3)	−0.006 (2)	0.002 (2)
C5	0.044 (3)	0.031 (2)	0.034 (2)	0.001 (2)	0.002 (2)	0.0024 (19)
C6	0.035 (3)	0.037 (3)	0.041 (2)	−0.006 (2)	0.003 (2)	−0.001 (2)
C7	0.042 (3)	0.033 (2)	0.036 (2)	−0.005 (2)	0.004 (2)	0.006 (2)
C8	0.034 (3)	0.040 (3)	0.031 (2)	−0.001 (2)	0.003 (2)	0.003 (2)
C9	0.032 (3)	0.049 (3)	0.037 (2)	0.001 (2)	0.003 (2)	0.004 (2)
C10	0.033 (3)	0.039 (3)	0.032 (2)	−0.004 (2)	0.007 (2)	−0.0005 (19)
C11	0.032 (3)	0.094 (5)	0.048 (3)	−0.006 (3)	0.009 (2)	0.015 (3)
C12	0.035 (3)	0.128 (6)	0.061 (4)	0.012 (4)	0.003 (3)	0.012 (4)
C13	0.044 (3)	0.083 (4)	0.045 (3)	0.004 (3)	−0.005 (3)	0.014 (3)
C14	0.043 (3)	0.049 (3)	0.039 (2)	0.009 (3)	0.005 (2)	0.005 (2)
C15	0.053 (3)	0.042 (3)	0.036 (2)	−0.006 (3)	0.008 (2)	0.009 (2)
C16	0.060 (4)	0.065 (4)	0.036 (2)	−0.024 (3)	−0.006 (3)	0.007 (3)
C17	0.059 (4)	0.147 (7)	0.070 (4)	0.016 (5)	−0.012 (4)	0.030 (5)
C18	0.047 (3)	0.043 (3)	0.045 (3)	−0.006 (3)	0.004 (3)	0.004 (2)
C19	0.073 (4)	0.055 (4)	0.059 (3)	−0.009 (3)	−0.020 (3)	−0.001 (3)
C20	0.050 (3)	0.042 (3)	0.038 (2)	0.001 (3)	−0.005 (2)	0.001 (2)
C21	0.084 (5)	0.058 (4)	0.051 (3)	0.027 (4)	0.000 (3)	−0.006 (3)
C22	0.059 (4)	0.066 (4)	0.083 (4)	0.019 (4)	−0.004 (4)	−0.004 (3)
C23	0.068 (4)	0.040 (3)	0.039 (2)	0.010 (3)	0.006 (3)	0.008 (2)
C24	0.047 (3)	0.058 (3)	0.043 (3)	−0.001 (3)	0.007 (2)	0.003 (2)
C25	0.051 (3)	0.046 (3)	0.031 (2)	−0.007 (3)	0.004 (2)	−0.004 (2)
C26	0.053 (4)	0.072 (4)	0.047 (3)	−0.003 (3)	0.014 (3)	0.003 (3)
C27	0.055 (4)	0.071 (4)	0.058 (3)	0.011 (3)	−0.001 (3)	0.004 (3)
C28	0.077 (5)	0.063 (4)	0.047 (3)	0.011 (4)	0.005 (3)	0.012 (3)
C29	0.060 (4)	0.044 (3)	0.044 (3)	−0.007 (3)	0.010 (3)	0.001 (2)
C30	0.048 (3)	0.034 (3)	0.037 (2)	−0.006 (2)	0.002 (2)	−0.012 (2)
N1	0.049 (3)	0.051 (3)	0.034 (2)	−0.006 (3)	0.003 (2)	−0.0093 (19)

### Geometric parameters (Å, º)

**Table:**

O1—C16	1.207 (7)	C12—H12A	0.9700
O2—C20	1.201 (6)	C12—H12B	0.9700
O3—C20	1.333 (6)	C13—C14	1.505 (8)
O3—C21	1.465 (7)	C13—C16	1.520 (9)
O4—C24	1.405 (6)	C13—C17	1.525 (8)
O4—C23	1.432 (6)	C14—H14A	0.9700
O5—N1	1.204 (6)	C14—H14B	0.9700
O6—N1	1.219 (6)	C15—C23	1.525 (8)
C1—C2	1.534 (7)	C15—C16	1.548 (8)
C1—C10	1.544 (6)	C15—H15A	0.9800
C1—H1A	0.9700	C17—H17A	0.9600
C1—H1B	0.9700	C17—H17B	0.9600
C2—C3	1.518 (9)	C17—H17C	0.9600
C2—H2A	0.9700	C18—H18A	0.9600
C2—H2B	0.9700	C18—H18B	0.9600
C3—C4	1.532 (8)	C18—H18C	0.9600
C3—H3A	0.9700	C19—H19A	0.9600
C3—H3B	0.9700	C19—H19B	0.9600
C4—C20	1.530 (7)	C19—H19C	0.9600
C4—C19	1.550 (8)	C21—C22	1.505 (9)
C4—C5	1.563 (6)	C21—H21A	0.9700
C5—C6	1.535 (6)	C21—H21B	0.9700
C5—C10	1.569 (7)	C22—H22A	0.9600
C5—H5A	0.9800	C22—H22B	0.9600
C6—C7	1.509 (7)	C22—H22C	0.9600
C6—H6A	0.9700	C23—H23A	0.9700
C6—H6B	0.9700	C23—H23B	0.9700
C7—C8	1.527 (7)	C24—C25	1.521 (7)
C7—H7A	0.9700	C24—H24A	0.9700
C7—H7B	0.9700	C24—H24B	0.9700
C8—C14	1.535 (7)	C25—C26	1.384 (8)
C8—C9	1.569 (6)	C25—C30	1.390 (7)
C8—C15	1.572 (7)	C26—C27	1.388 (9)
C9—C11	1.551 (8)	C26—H26A	0.9300
C9—C10	1.561 (7)	C27—C28	1.376 (9)
C9—H9A	0.9800	C27—H27A	0.9300
C10—C18	1.542 (7)	C28—C29	1.387 (9)
C11—C12	1.534 (9)	C28—H28A	0.9300
C11—H11A	0.9700	C29—C30	1.378 (7)
C11—H11B	0.9700	C29—H29A	0.9300
C12—C13	1.543 (8)	C30—N1	1.485 (7)
			
C20—O3—C21	116.6 (4)	C13—C14—H14A	110.8
C24—O4—C23	112.6 (4)	C8—C14—H14A	110.8
C2—C1—C10	113.8 (5)	C13—C14—H14B	110.8
C2—C1—H1A	108.8	C8—C14—H14B	110.8
C10—C1—H1A	108.8	H14A—C14—H14B	108.9
C2—C1—H1B	108.8	C23—C15—C16	108.6 (4)
C10—C1—H1B	108.8	C23—C15—C8	117.6 (5)
H1A—C1—H1B	107.7	C16—C15—C8	103.5 (4)
C3—C2—C1	110.7 (5)	C23—C15—H15A	108.9
C3—C2—H2A	109.5	C16—C15—H15A	108.9
C1—C2—H2A	109.5	C8—C15—H15A	108.9
C3—C2—H2B	109.5	O1—C16—C13	127.7 (6)
C1—C2—H2B	109.5	O1—C16—C15	123.6 (6)
H2A—C2—H2B	108.1	C13—C16—C15	108.7 (4)
C2—C3—C4	113.6 (4)	C13—C17—H17A	109.5
C2—C3—H3A	108.8	C13—C17—H17B	109.5
C4—C3—H3A	108.8	H17A—C17—H17B	109.5
C2—C3—H3B	108.8	C13—C17—H17C	109.5
C4—C3—H3B	108.8	H17A—C17—H17C	109.5
H3A—C3—H3B	107.7	H17B—C17—H17C	109.5
C20—C4—C3	109.2 (4)	C10—C18—H18A	109.5
C20—C4—C19	105.6 (5)	C10—C18—H18B	109.5
C3—C4—C19	108.2 (5)	H18A—C18—H18B	109.5
C20—C4—C5	115.6 (4)	C10—C18—H18C	109.5
C3—C4—C5	108.6 (4)	H18A—C18—H18C	109.5
C19—C4—C5	109.4 (4)	H18B—C18—H18C	109.5
C6—C5—C4	117.9 (4)	C4—C19—H19A	109.5
C6—C5—C10	110.8 (4)	C4—C19—H19B	109.5
C4—C5—C10	114.5 (4)	H19A—C19—H19B	109.5
C6—C5—H5A	103.9	C4—C19—H19C	109.5
C4—C5—H5A	103.9	H19A—C19—H19C	109.5
C10—C5—H5A	103.9	H19B—C19—H19C	109.5
C7—C6—C5	110.7 (4)	O2—C20—O3	123.1 (5)
C7—C6—H6A	109.5	O2—C20—C4	123.9 (5)
C5—C6—H6A	109.5	O3—C20—C4	112.9 (4)
C7—C6—H6B	109.5	O3—C21—C22	112.0 (5)
C5—C6—H6B	109.5	O3—C21—H21A	109.2
H6A—C6—H6B	108.1	C22—C21—H21A	109.2
C6—C7—C8	114.0 (4)	O3—C21—H21B	109.2
C6—C7—H7A	108.8	C22—C21—H21B	109.2
C8—C7—H7A	108.8	H21A—C21—H21B	107.9
C6—C7—H7B	108.8	C21—C22—H22A	109.5
C8—C7—H7B	108.8	C21—C22—H22B	109.5
H7A—C7—H7B	107.7	H22A—C22—H22B	109.5
C7—C8—C14	111.6 (4)	C21—C22—H22C	109.5
C7—C8—C9	109.5 (4)	H22A—C22—H22C	109.5
C14—C8—C9	106.5 (4)	H22B—C22—H22C	109.5
C7—C8—C15	115.8 (4)	O4—C23—C15	108.0 (4)
C14—C8—C15	101.3 (4)	O4—C23—H23A	110.1
C9—C8—C15	111.7 (4)	C15—C23—H23A	110.1
C11—C9—C10	113.4 (4)	O4—C23—H23B	110.1
C11—C9—C8	110.1 (4)	C15—C23—H23B	110.1
C10—C9—C8	118.3 (4)	H23A—C23—H23B	108.4
C11—C9—H9A	104.5	O4—C24—C25	108.3 (5)
C10—C9—H9A	104.5	O4—C24—H24A	110.0
C8—C9—H9A	104.5	C25—C24—H24A	110.0
C18—C10—C1	109.9 (4)	O4—C24—H24B	110.0
C18—C10—C9	113.1 (4)	C25—C24—H24B	110.0
C1—C10—C9	107.0 (4)	H24A—C24—H24B	108.4
C18—C10—C5	111.4 (4)	C26—C25—C30	116.3 (5)
C1—C10—C5	107.9 (4)	C26—C25—C24	119.8 (5)
C9—C10—C5	107.3 (4)	C30—C25—C24	123.8 (5)
C12—C11—C9	112.5 (5)	C25—C26—C27	121.3 (6)
C12—C11—H11A	109.1	C25—C26—H26A	119.3
C9—C11—H11A	109.1	C27—C26—H26A	119.3
C12—C11—H11B	109.1	C28—C27—C26	120.7 (6)
C9—C11—H11B	109.1	C28—C27—H27A	119.6
H11A—C11—H11B	107.8	C26—C27—H27A	119.6
C11—C12—C13	112.9 (5)	C27—C28—C29	119.5 (6)
C11—C12—H12A	109.0	C27—C28—H28A	120.3
C13—C12—H12A	109.0	C29—C28—H28A	120.3
C11—C12—H12B	109.0	C30—C29—C28	118.5 (5)
C13—C12—H12B	109.0	C30—C29—H29A	120.8
H12A—C12—H12B	107.8	C28—C29—H29A	120.8
C14—C13—C16	102.3 (5)	C29—C30—C25	123.6 (5)
C14—C13—C17	116.0 (6)	C29—C30—N1	115.2 (5)
C16—C13—C17	113.3 (5)	C25—C30—N1	121.2 (5)
C14—C13—C12	108.0 (4)	O5—N1—O6	124.3 (5)
C16—C13—C12	105.7 (6)	O5—N1—C30	118.5 (5)
C17—C13—C12	110.7 (5)	O6—N1—C30	117.2 (5)
C13—C14—C8	104.7 (5)		
			
C10—C1—C2—C3	56.0 (6)	C9—C8—C14—C13	−72.8 (5)
C1—C2—C3—C4	−56.2 (6)	C15—C8—C14—C13	44.0 (5)
C2—C3—C4—C20	−72.3 (6)	C7—C8—C15—C23	−30.1 (6)
C2—C3—C4—C19	173.2 (5)	C14—C8—C15—C23	90.7 (5)
C2—C3—C4—C5	54.5 (6)	C9—C8—C15—C23	−156.3 (4)
C20—C4—C5—C6	−64.0 (6)	C7—C8—C15—C16	−149.8 (4)
C3—C4—C5—C6	172.9 (4)	C14—C8—C15—C16	−29.0 (5)
C19—C4—C5—C6	54.9 (6)	C9—C8—C15—C16	84.0 (4)
C20—C4—C5—C10	69.0 (6)	C14—C13—C16—O1	−157.8 (6)
C3—C4—C5—C10	−54.0 (5)	C17—C13—C16—O1	−32.2 (9)
C19—C4—C5—C10	−172.0 (4)	C12—C13—C16—O1	89.2 (7)
C4—C5—C6—C7	−162.9 (4)	C14—C13—C16—C15	21.6 (5)
C10—C5—C6—C7	62.4 (5)	C17—C13—C16—C15	147.2 (5)
C5—C6—C7—C8	−59.3 (5)	C12—C13—C16—C15	−91.4 (5)
C6—C7—C8—C14	166.3 (4)	C23—C15—C16—O1	58.8 (7)
C6—C7—C8—C9	48.7 (5)	C8—C15—C16—O1	−175.5 (5)
C6—C7—C8—C15	−78.6 (5)	C23—C15—C16—C13	−120.7 (5)
C7—C8—C9—C11	−178.2 (5)	C8—C15—C16—C13	5.0 (5)
C14—C8—C9—C11	61.1 (6)	C21—O3—C20—O2	5.5 (8)
C15—C8—C9—C11	−48.6 (6)	C21—O3—C20—C4	−178.2 (5)
C7—C8—C9—C10	−45.6 (6)	C3—C4—C20—O2	−9.0 (8)
C14—C8—C9—C10	−166.3 (4)	C19—C4—C20—O2	107.1 (6)
C15—C8—C9—C10	84.0 (5)	C5—C4—C20—O2	−131.8 (6)
C2—C1—C10—C18	68.0 (6)	C3—C4—C20—O3	174.7 (5)
C2—C1—C10—C9	−168.8 (5)	C19—C4—C20—O3	−69.2 (5)
C2—C1—C10—C5	−53.6 (6)	C5—C4—C20—O3	51.9 (6)
C11—C9—C10—C18	56.8 (6)	C20—O3—C21—C22	−79.3 (7)
C8—C9—C10—C18	−74.3 (6)	C24—O4—C23—C15	−178.5 (4)
C11—C9—C10—C1	−64.3 (6)	C16—C15—C23—O4	53.3 (6)
C8—C9—C10—C1	164.6 (4)	C8—C15—C23—O4	−63.6 (6)
C11—C9—C10—C5	−180.0 (4)	C23—O4—C24—C25	166.8 (4)
C8—C9—C10—C5	49.0 (5)	O4—C24—C25—C26	−22.9 (7)
C6—C5—C10—C18	69.1 (5)	O4—C24—C25—C30	160.5 (5)
C4—C5—C10—C18	−67.2 (5)	C30—C25—C26—C27	−0.3 (9)
C6—C5—C10—C1	−170.3 (4)	C24—C25—C26—C27	−177.1 (5)
C4—C5—C10—C1	53.4 (5)	C25—C26—C27—C28	1.0 (10)
C6—C5—C10—C9	−55.2 (5)	C26—C27—C28—C29	−1.8 (10)
C4—C5—C10—C9	168.5 (4)	C27—C28—C29—C30	2.1 (9)
C10—C9—C11—C12	177.6 (5)	C28—C29—C30—C25	−1.5 (8)
C8—C9—C11—C12	−47.4 (7)	C28—C29—C30—N1	178.5 (5)
C9—C11—C12—C13	45.6 (8)	C26—C25—C30—C29	0.6 (8)
C11—C12—C13—C14	−57.4 (8)	C24—C25—C30—C29	177.3 (5)
C11—C12—C13—C16	51.6 (7)	C26—C25—C30—N1	−179.3 (5)
C11—C12—C13—C17	174.6 (7)	C24—C25—C30—N1	−2.7 (7)
C16—C13—C14—C8	−40.9 (5)	C29—C30—N1—O5	37.0 (6)
C17—C13—C14—C8	−164.7 (5)	C25—C30—N1—O5	−143.0 (5)
C12—C13—C14—C8	70.4 (6)	C29—C30—N1—O6	−141.5 (5)
C7—C8—C14—C13	167.8 (4)	C25—C30—N1—O6	38.4 (7)
